# Improving Planetary Rover Attitude Estimation via MEMS Sensor Characterization

**DOI:** 10.3390/s120202219

**Published:** 2012-02-15

**Authors:** Javier Hidalgo, Pantelis Poulakis, Johan Köhler, Jaime Del-Cerro, Antonio Barrientos

**Affiliations:** 1 Centro de Automática y Robótica, UPM-CSIC, José Gutiérrez Abascal 2, Madrid 28006, Spain; E-Mails: j.cerro@upm.es (J.D.-C.) antonio.barrientos@upm.es (A.B.); 2 Automation and Robotics Section, ESA/ESTEC, Noordwijk 2200 AG, The Netherlands; E-Mail: pantelis.poulakis@esa.int

**Keywords:** micro-electro-mechanical systems (MEMS), inertial measurement unit (IMU), inertial navigation system (INS), sensor characterization, sensor fusion, attitude estimation, planetary rover

## Abstract

Micro Electro-Mechanical Systems (MEMS) are currently being considered in the space sector due to its suitable level of performance for spacecrafts in terms of mechanical robustness with low power consumption, small mass and size, and significant advantage in system design and accommodation. However, there is still a lack of understanding regarding the performance and testing of these new sensors, especially in planetary robotics. This paper presents what is missing in the field: a complete methodology regarding the characterization and modeling of MEMS sensors with direct application. A reproducible and complete approach including all the intermediate steps, tools and laboratory equipment is described. The process of sensor error characterization and modeling through to the final integration in the sensor fusion scheme is explained with detail. Although the concept of fusion is relatively easy to comprehend, carefully characterizing and filtering sensor information is not an easy task and is essential for good performance. The strength of the approach has been verified with representative tests of novel high-grade MEMS inertia sensors and exemplary planetary rover platforms with promising results.

## 1. Introduction

Inertial sensors play a prominent role in the navigation of a wide range of vehicles, aiming at improving the onboard attitude estimation and enhancing the overall onboard localization capabilities. In particular for the case of planetary rovers, robust attitude estimation is critical for numerous operations, such as pointing the antenna for direct-to-earth or orbiter communications, controlling the body posture for maximum power generation by the solar panels and precision placement of instruments on scientific targets.

There are several areas on which the navigation and robotics research communities have focused their efforts over the years in order to integrate and improve the performance of an inertial measurements unit (IMU) onboard aerial/terrestrial/underwater vehicles. Methods for characterizing inertial sensor errors are discussed in [1–3]. Specifically, in [2], El-Sheimy *et al*. provided a thorough methodology of using the Allan Variance method to characterize the noise terms of inertial sensor data and established a relationship between the Allan Variance and the noise Power Spectral Density (PSD). However, a direct application is not given. Several approaches have also been proposed for modelling inertial sensors and their error behaviour. More specifically, the work in [4] introduces a procedure for modeling low-cost sensors and in [5] a detailed Allan variance analysis is provided. In addition to this, recent works in sensor fusion such as [6,7] build upon the idea that correctly incorporated inertial data in an intelligence and precise manner is still a challenge in the data fusion domain.

This paper presents elements that are considered to be missing in the literature on the topic, which is a systematic End-to-End (E2E) approach for incorporating an inertial sensor in the localization scheme of a mobile robot. All the steps of utilizing an inertial sensor for attitude estimation are addressed in a unified methodology, starting from the characterization of the dominant sensor errors, then deriving a suitable sensor error model, subsequently designing an adequate Kalman filter and finally implementing an onboard sensor fusion scheme. The complete chain of the steps and performance of the algorithms is verified by implementation onboard planetary rover breadboards developed by the European Space Agency (ESA) and tested in the Planetary Utilization Testbed (PUTB) of the European Space Research and Technology Centre (ESTEC). The presented E2E approach offers a sufficiently generic methodology to be applied to any inertial sensor, addressing for the first time in a systematic way all the steps involved from choosing an inertial sensor up to designing an onboard sensor fusion scheme, while proposing a high performance attitude reference system for planetary rovers fully based on tactical grade MEMS IMU.

**Figure 1.**
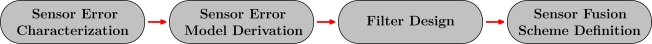
Schematic representation of the End-to-End Approach.

A schematic overview of the proposed E2E methodology can be seen in Figure 1. In this paper every block of Figure 1 is presented in a separate section. In the beginning of each section the applicability of each block is critically discussed and the respective theory is elaborated upon. In the first two sections on sensor error characterization and modelling the theory is directly applied to a modern prototype IMU based on MEMS components, the Imego IMT30.

Section 2 discusses the sensor error characterization method based on the Allan Variance technique and analyzes the results of this method on the IMT30 prototype IMU. Section 3 presents the proposed sensor error modeling approach and derives the model of IMT30. The filtering technique based on Unscented Kalman Filter as well as the rover’s attitude kinematics and the state vector are analyzed in Section 4. The proposed architecture for the rover’s attitude estimation scheme is explained in Section 5. Subsequently, Section 6 describes the experimental testing and results of the presented E2E approach onboard a rover platform. Finally, the paper concludes with a discussion on the results.

## 2. Sensor Error Characterization

Characterizing the inertial sensor errors can be a intensive process due to the number of tests and the statistical analysis required. The output of inertial sensors are influenced by a wide range of error sources. They can be classified as deterministic and stochastic errors. Deterministic errors are disturbances in which no randomness is involved and are likely to be temperature dependent in MEMS technology. Stochastic errors are disturbances in the signal introduced by random processes which are the counterpart to deterministic processes. There is some indeterminacy in its future evolution affecting the signal by noise in the sensor itself or in other electronic equipment (transducer). Moreover dedicated testing facilities are desirable, such as a seismic block and a rate table, which are not widely available in laboratories and the access to them can complicate the process. In most of the cases sensors manufacturers do not provide reliable sensor error analysis in order to be included in the filter design and thus the characterization process is often mandatory, especially considering new MEMS sensors development as that one tested in this activity.

The correlation-function approach and its corresponding transform in the frequency-domain, the Power Spectral Density (PSD), represent the basics of characterization and modeling of stochastic errors, having advantages and limitations. The Allan variance has gained importance due to the computational simplicity and quick adaptation to noise characterization of a variety of sensors. There are also few limitations in the mapping from the Allan variance to the spectrum however the analysis remains useful. This work applies the Allan variance to identify and characterize the type and magnitudes of the most dominant random errors.

### 2.1. Allan Variance Technique

**Figure 2.**
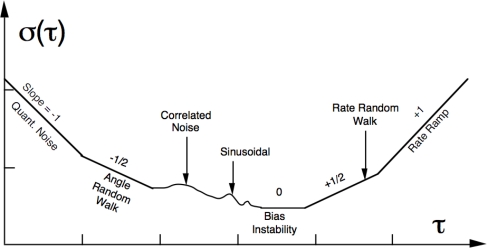
Allan variance plot from the IEEE Std 952–1997 for typical data analysis [8]. The cluster time of length *τ* could take different units in time (e.g., microseconds, seconds, minutes or hours) as well as *σ*(*τ*), *i.e.*, angular velocity (e.g., *rad/s* or *°/h*) for gyros or acceleration (e.g., *g* or *m/s*^2^) in case of accelerometers, depending on sensor type.

The Allan variance *σ*^2^(*τ*) provides direct information on the type and magnitude of various noise terms by splitting static measurement values into clusters. A statistical variance is computed among clusters of same size by taking collected values. The Allan variance of a cluster time of length *τ* is estimated as:

(1) $$\sigma^{2}(\tau )=\frac{1}{2(N-2n)}\sum_{k=1}^{N-2n}{\left[{\bar{\Omega }}_{k+1}(t)-{\bar{\Omega }}_{k}(t)\right]}^{2}$$

where *N* is the total amount of sensor values and Ω̄(*t*) represents the cluster average value of the output for a cluster which starts from the *k^th^* data point and contains *n* data points depending on the length of *τ*. The Allan variance is plotted in a log-log graph as the standard deviation *versus* the cluster time *τ* in order to characterize the different noise terms (see Figure 2). Modeling of stochastic errors requires identifying its PSD function. There is a unique relationship between the Allan variance (time domain) and the PSD (frequency domain) of the random process defined by:

(2) $$\sigma^{2}(\tau )=4\int_{0}^{\infty }S_{\Omega }(f)\frac{\sin^{4}(\pi f\tau )}{{\left(\pi f\tau \right)}^{2}}\mathrm{df}$$

where *S*_Ω_(*f*) is the PSD of the random process. The different random processes are characterized at various frequencies varying *τ* by using fitting methods. Further explanation of typical error presented in inertial sensors can be found in [8]. The application of the technique using the prototype of an IMU is shown in the following.

### 2.2. IMT30 Characterization

The Automation & Robotics Section of ESA procured the prototype IMT30 IMU from the Imego [9] Institute of Micro and Nanotechnology in order to assess its performance for planetary rover attitude estimation. The IMT30, though not space qualified, is a solid state six degrees of freedom IMU prototype based on MEMS components with small size and weight. Inside the case, the IMU is comprised of 3 accelerometers and 3 gyroscopes with electronics, temperature sensors and a Field-programmable Gate Array (FPGA) onboard.

**Figure 3.**
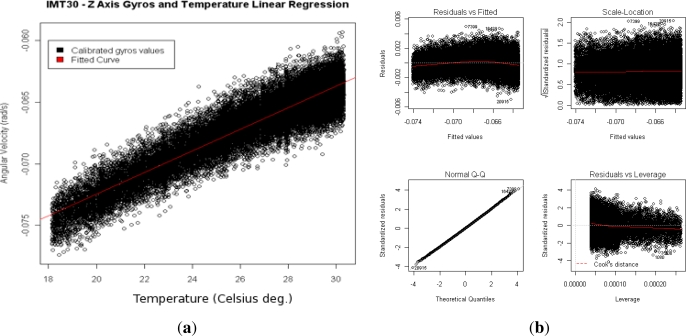
Thermal drift regression for Z gyros axis. **(a)** Temperature correlated bias and linear regression curve; **(b)** Residual information of the fitted curve.

As mentioned before, MEMS inertial sensor are likely influenced by the temperature. Temperature variation directly affects sensor bias and therefore will influence in the final analysis. Thermal drift needs to be identified and compensated in order to precisely characterize the stochastic errors underlying the sensor signal. Thermal drift for the gyroscope of the IMT30 was performed and analyzed, and is presented in Table 1 and shown in Figure 3. A simple linear regression perfectly fits the thermal drift.

#### Test Setup

**Figure 4.**
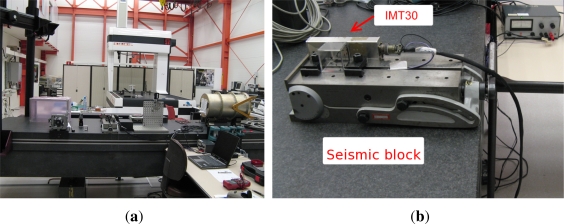
Metrology laboratory at ESA/ ESTEC. **(a)** General view with the seismic block in the foreground; **(b)** IMT30 on the seismic block while testing.

Five tests were conducted at the Metrology Laboratory of ESTEC, which is a semi-clean room equipped with an anti-vibration table (seismic block) in order to perform accurate static measurements (see Figure 4). Four hours of static data were collected per test on the seismic block by an external PC, with 16 bits resolution. According to Papoulis in [10], to characterize a signal the sampling frequency should be at least six times the bandwidth of the sensor. The IMT30 gyros have a bandwidth of 100 Hz and the acquisition frequency was set to 976 Hz by the manufacturer. The data were subsequently analyzed using the Allan variance technique [8].

#### Allan Variance Results

**Figure 5.**
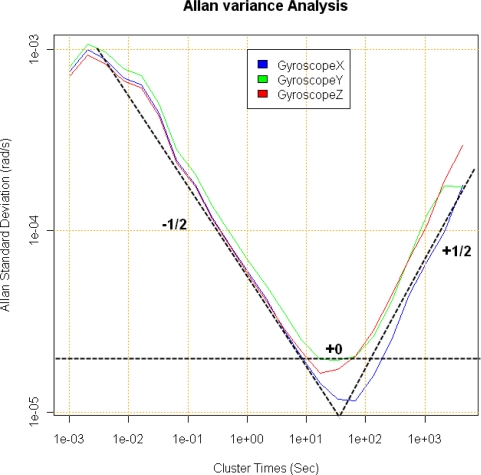
Allan variance analysis for IMT30 gyroscopes.

The results of applying the Allan variance to the recorded data is depicted in the plot of Figure 5. The graph shows that the angle random walk is the dominant error for short cluster times, where the curve fits a straight line of slope *−*(1/2). The numerical value of angle random walk *N* (see Table 2) can be obtained by reading the slope line at *τ* = 1 and working out the *N* value from the angle random walk equation in the Allan variance (see Table 3). Also a straight line of slope +(1/2) fits for the long cluster times part of the plot. The magnitude *K* (see Table 4) can be obtained by reading the slope line at *τ* = 3 and obtaining the *K* coefficient from the corresponding equation in Table 3. The center of the curve shows a small flat part identified by a zero slope. It characterizes a bias instability *B*, which represents the best stability of the run. The conventional unit for the bias instability is *^°^/h* [11] and gives the sensor grade being categorized as *tactical-grade* (see Table 5). Further information about inertial sensor categories can be found in [12].

## 3. Sensor Modeling

### 3.1. General

Inertial Navigation Systems (INS) performance can be significantly enhanced by the incorporation of a sensor error model in the state estimation process of a Kalman filter. Here a sensor model is proposed that is balanced between accuracy and complexity and composed of deterministic and stochastic sensor errors. Some deterministic errors, like the thermal drift, are directly compensated after the sensor read-out, others, like the scale factor and the misalignment, are included in the model. It should also be noted that the proposed sensor model covers the commonly encountered errors in inertial sensors but does not include g-sensitive drift, scale factor asymmetry or other errors which depend on the design of a particular sensor. The sensor model is given by:

(3) $$\tilde{\omega }(t)=(I_{3\times 3}+M(\mathrm{SF},\mathrm{MA}))\omega (t)+n_{s}(t)$$

where ***ω̃***(*t*) is the 3 × 1 continuous time measured sensor value (*i.e.*, [*p, q, r*]*^T^* coming from gyros), *M* is the deterministic errors matrix corresponding to the misalignment (*MA*) and scale factor (*SF*) assumed to be temperature independent and ***n****_s_*(*t*) is the stochastic noise process.

Modeling a stochastic error requires identifying its PSD function in order to incorporate it in ***n****_s_*(*t*). Stochastic errors are modeled as a linear time invariant system, by having the knowledge of the PSD function of the output and assuming unit white noise input. The associated PSD function is used to shape a stationary input into a given spectral function. It is known as the shaping filter approach [13] and the spectral function is unique for each stochastic noise type. The emphasis of the unified model is to combine the equations for the different types of noise described in Table 3. Depending on the kind of noise presented, there are certain limitations in the definition of the linear equations of the shaping filter. It is referred to as the quantization noise because Kalman Filter theory only performs on differential equations driven by white noise, and thus quantization noise will have a noise source which is the derivative of the white noise. Under this situation different consideration should be taken using acceptable approximations, like those ones detailed in [14,15]. Although this limitation affects the model design, common stochastic errors presented in inertial sensor can be directly incorporated in the model.

### 3.2. IMT30 Gyroscope Model

The IMT30 error characterization depicts the angle random walk and the rate angle random walk as the dominant stochastic errors for gyros. The first error is affected by white noise while the second one affects the bias instability, or gyros drift. The PSD of the random walk is defined by *N*^2^ (see Table 3) and it can be directly incorporated into the model. The PSD of the rate angle random walk is defined by *K*^2^*/s*^2^ and it is incorporated in the model by *K/s*, replacing *jω* by *s* in Laplace domain. Consequently, the derived gyros model is:

(4a) $$\tilde{\omega }(t)=(I_{3\times 3}+M(\mathrm{SF},\mathrm{MA}))\omega (t)+\beta_{\omega }(t)+n_{\mathrm{rw}}(t)$$

(4b) $${\dot{\beta }}_{\omega }(t)=n_{\mathrm{rrw}}(t)$$

where ***ω̃***(*t*) is the sensor readout, ***ω***(*t*) is the ideal value, ***β****_ω_*(*t*) is the bias and ***n****_rω_*(*t*) and ***n****_rrω_*(*t*) are independent zero-mean Gaussian white noise of the characterized stochastic processes defined with

(5a) $$E\{n_{r\omega }(t)n_{r\omega }^{T}(\tau )\}=I_{3\times 3}N^{2}\delta (t-\tau )$$

(5b) $$E\{n_{\mathrm{rr}\omega }(t)n_{\mathrm{rr}\omega }^{T}(\tau )\}=I_{3\times 3}K^{2}\delta (t-\tau )$$

where *E* denotes expectation, *δ* (*t − τ*) is the Dirac delta function, *N* and *K* are the angle random walk and rate random walk coefficients of Tables 2 and 4 in $\mathrm{rad}/\sqrt{s}$ and $\mathrm{rad}/s/\sqrt{s}$ respectively.

## 4. Unscented Filtering

Filtering is the next essential step and aims at the estimation of the defined state from noise and error affected sensor readings. In the frame of the presented methodology for rover attitude estimation, the authors propose the use of the Unscented Kalman Filter (UKF) due to the clear advantages in propagating the state estimation of the system over the Extended Kalman Filter (EKF). In the UKF, given a *n × n* covariance matrix *P*, a set of 2*n* sigma points are generated from the columns of the matrices $\pm \sqrt{[n+\lambda ]P}$, where λ ≥ 0. The sigma points completely capture the true mean and covariance of the prior state estimation and when propagated through the nonlinear system, the UKF captures the posterior mean and covariance more accurately than the conventional EKF [7,16,17].

### 4.1. Rover Attitude Kinematics

It is proposed that the rover attitude is expressed by a quaternion as a four parameters representation of a transformation matrix. Quaternions do not involve transcendental functions, and thus, kinematics are bilinear and free of singularities. The quaternion is defined by:

(6) $$q\equiv {\left[\varrho^{T}q_{4}\right]}^{T}$$

with ***ϱ*** = [*q*_1_, *q*_2_, *q*_3_]*^T^* = *ê* sin(*ϑ/*2) and *q*_4_ = cos(*ϑ/*2) where *ê* is the axis of rotation and *ϑ* the angle of rotation. The attitude kinematics equation is given in the continuous time domain by:

(7) $$\dot{q}(t)=\frac{1}{2}\Xi [q(t)]\omega (t)$$

where ***ω***(*t*) is the 3 × 1 angular rate vector and

(8) $$\Xi =\left[\begin{array}{c} q_{4}I_{3\times 3}+[\varrho \times ]\\ -\varrho^{T} \end{array}\right]$$

The delta quaternion is usually denoted in the literature by ***δ****q ≡* [***δϱ****^T^ δq*_4_]*^T^* which will be used later in the propagation. The delta quaternion represents small variation of rovers attitude in each filter iteration and it can be expressed as Modified Rodrigues Parameters (MRP) since it will never reach the singularity in practice (at 360° for MRP). The relation between Modified Rodrigues Parameters and delta quaternion is given by:

(9) $$\delta p\equiv f[\delta \varrho /(a+\delta q_{4})]$$

where *a* is a parameter between 0 and 1 and *f* is a scale factor. When *f* = *a* = 1 the formula gives the standard three component representation for the MRP [16]. It should also be noted here that MRP provide a very compact notation and a multiplicative approach, to avoid quaternion renormalization, deals with successive multiplication of delta quaternion updates after propagating through the system.

### 4.2. State Vector

The equations in this section refer to the discrete time domain. The tilde (*ã*) notation indicates magnitude measurement, the hat (*â*) refers to magnitude estimation and the plus (^+^) and minus (*−*) superscripts indicate post- and pre-updates respectively. Using Kalman formulation, we define the state-vector of the system in Equation (10) by using the MRP and by incorporating the derived sensor model.

(10) $${\hat{x}}_{k}^{+}={\left[\delta {\hat{p}}_{k}^{+},{\hat{\beta }}_{k}^{+}\right]}^{T}$$

$\delta {\hat{p}}_{k}^{+}$ represents the delta quaternion using the MRP and ${\hat{\beta }}_{k}^{+}$ is the estimate gyros bias of the model. Given a estimate ${\hat{\beta }}_{k}^{+}$ of the gyros model, the post update angular velocity and propagated gyros bias follows:

(11a) $${\hat{\omega }}_{k}^{+}={\tilde{\omega }}_{k}-M{\tilde{\omega }}_{k}-C_{g}^{b}\omega_{i/e}^{g}-{\hat{\beta }}_{k}^{+}$$

(11b) $${\hat{\beta }}_{k+1}^{-}={\hat{\beta }}_{k}^{+}$$

where $C_{g}^{b}$ is the transformation attitude matrix from geographic frame to body frame, $\omega_{i/e}^{g}$ is the angular earth rotation vector in the geographic frame and it is expressed as

(12) $$\omega_{i/e}^{g}={\left[\omega_{i/e}\;\cos(\Phi ),0,\omega_{i/e}\;\sin(\Phi )\right]}^{T}$$

where *ω_i/e_* is the Earth angular velocity equal to 7.292115 × 10*^−^*^5^*rad/s* according to the WGS-84 Earth reference model and Φ the latitude [18].

The propagated quaternion can be described by the discrete time attitude kinematics Equation (7) as a function of the post-update estimate rover angular velocity ${\hat{\omega }}_{k}^{+}$ and the quaternion ${\hat{q}}_{k}^{+}$,

(13) $${\hat{q}}_{k+1}^{-}=\Omega ({\hat{\omega }}_{k}^{+}){\hat{q}}_{k}^{+}$$

with

$$\Omega \left({\hat{\omega }}_{k}^{+}\right)=\left[\begin{array}{cc} K_{k} & {\hat{\psi }}_{k}^{+}\\ -{\hat{\psi }}_{k}^{+} & \text{cos}(0.5\left\|{\hat{\omega }}_{k}^{+}\right\|\Delta t) \end{array}\right]$$

where $K_{k}\equiv \text{cos}(0.5\left\|{\hat{\omega }}_{k}^{+}\right\|\Delta t)I_{3\times 3}-\left[{\hat{\psi }}_{k}^{+}\times \right]$, ${\hat{\psi }}_{k}^{+}\equiv \sin(0.5\left\|{\hat{\omega }}_{k}^{+}\right\|\Delta t){\hat{\omega }}_{k}^{+}/\left\|{\hat{\omega }}_{k}^{+}\right\|$ and Δ*t* is the sampling interval of the gyro. The discrete process noise covariance matrix is given [16] by

(14) $$Q_{k}=\left[\begin{array}{cc} (N^{2}\Delta t+\frac{1}{3}K^{2}\Delta t^{3})I_{3\times 3} & -\left(\frac{1}{2}K^{2}\Delta t^{2}\right)I_{3\times 3}\\ -\left(\frac{1}{2}K^{2}\Delta t^{2}\right)I_{3\times 3} & (K^{2}\Delta t)I_{3\times 3} \end{array}\right]$$

## 5. Sensor Fusion Scheme

In the case of robots and vehicles, the sensor fusion scheme design is highly dependent on the type and amount of sensors, on the type of vehicle (aerial, terrestrial, underwater, *etc*.) and the operational conditions (e.g., high/low dynamics). In this section a scheme is presented for attitude estimation based only on an inertial sensor with a planetary rover as the target platform. Though planetary rovers have low dynamics, the principles of the design can be applicable to other robotic vehicles as well.

**Figure 6.**
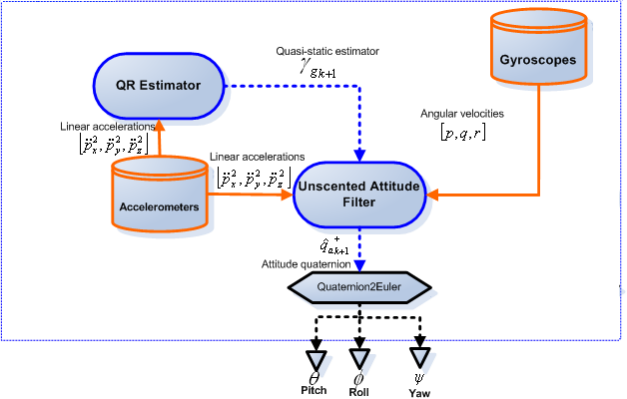
Sensor Fusion scheme on board the rover.

The architecture of the proposed sensor fusion scheme can be seen in Figure 6, where both the accelerometers and the gyros of the IMU are utilized and separate Kalman filters are implemented. The Unscented Attitude Filter (UAF) uses a quaternion based on Equation (10) and a system model based on Equation (13) for the estimation of the rover’s pitch, roll and yaw angles. It utilizes the accelerometers as inclinometers to correct the pitch and roll estimations in the correction step of the filter. A Quasi-static Regime Estimator (QR) similar to implementations in [6,19] is incorporated in the scheme. It estimates the dynamic/static regime of the rover by matching the measured gravity vector to the one already pre-programmed in the algorithm (theoretical value based on rover location) and thus gives an indication as to whether the accelerometer readings are reliable for the UAF correction step. QR system model is defined to estimate the instantaneous gravity magnitude as:

(15) $${\hat{x}}_{\mathrm{gk}}^{+}={\hat{x}}_{\mathrm{gk}}^{-}+K_{\mathrm{gk}}[a_{\mathrm{gk}}-{\hat{x}}_{\mathrm{gk}}^{-}]$$

where *x̂*_*gk*_ is the gravity estimation, *K*_*gk*_ the filter gain and $a_{\mathrm{gk}}=\sqrt{{\ddot{x}}_{k}+{\ddot{y}}_{k}+{\ddot{z}}_{k}}$. The accelerometer values are considered as three independent Gaussian random variables. The boolean variable γ_*gk*+1_ of the QR informs to the UAF how to combine both signals in a dynamic mode, by connecting/disconnecting accelerometers data in the correction step of the UAF filter. Therefore, when high accelerations are sensed on rover platform the QR informs to the UAF (via the γ_*gk*+1_ boolean value) of not including the pitch and roll estimation from the gravity vector.

## 6. Experimental Testing and Results

### 6.1. Experimental Test Setup

**Figure 7.**
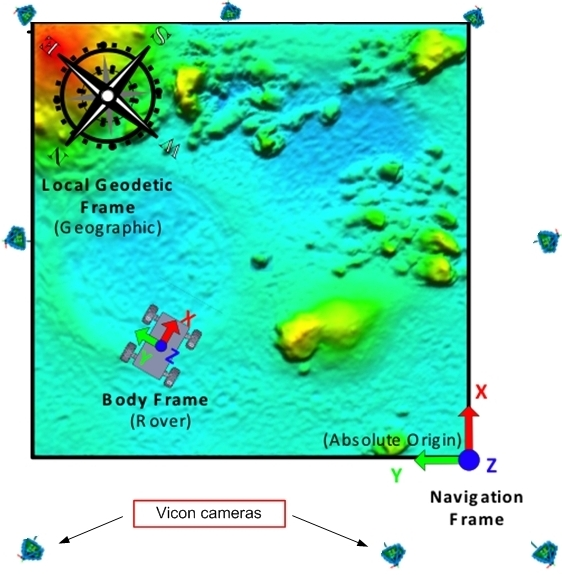
Coordinate frames and Vicon cameras deployment for the experimental setup in the PUTB.

**Figure 8.**
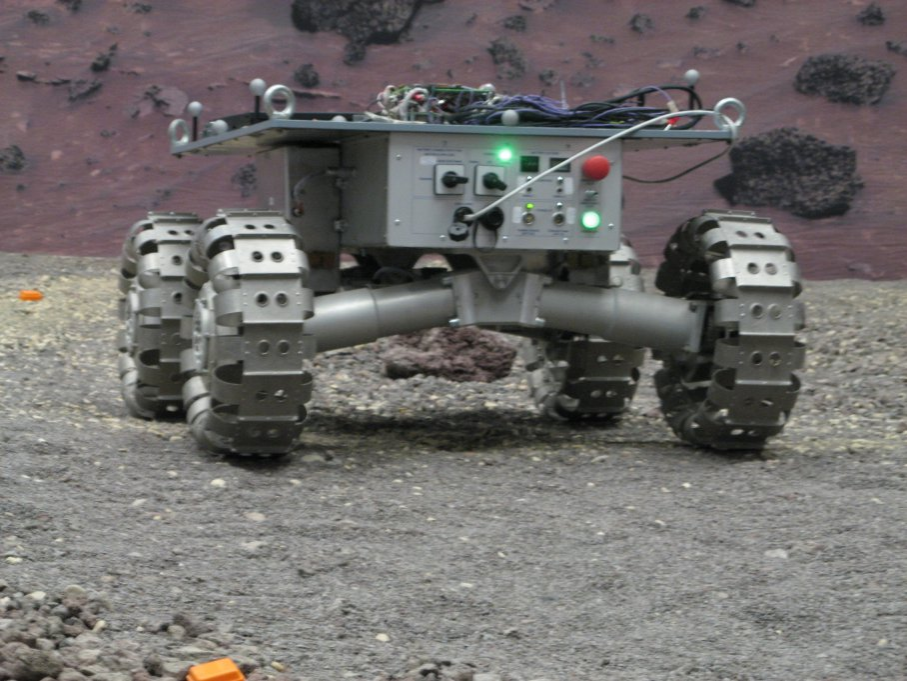
Lunar Rover Model (LRM) rover of ESA while performing a test in the PUTB.

The tests were carried out at the ESTEC PUTB, a 9 m × 9 m testbed that resembles a planetary surface (see Figure 7). Around the PUTB a set of eight infrared emitting and sensing cameras are mounted to the walls, which sense reflective markers mounted on rover platform. These cameras are part of the Vicon [20] system which can deduct and track position and orientation of objects equipped with such reflective markers. The precision in the attitude measurement precision of the Vicon system in the PUTB setup is in the order of 0.1°–0.2°. A planetary rover breadboard, the Lunar Rover Model (LRM) developed by ESA for engineering investigations of locomotion capabilities, was used as a representative platform to mount the IMT30 IMU for the tests (see Figure 8).

The software onboard the rover is in charge of obtaining raw values directly from the IMU. Afterwards, those values are calibrated and compensated using temperature values. Since planetary rovers do not have high dynamics the values are low-pass filtered at 10 Hz. Finally, the sensor fusion scheme runs in the last stage of the process, filtering and combining inertial information. Four test trajectories were implemented:

- Straight line trajectory of 5 m, on flat ground. Rover speed at 1 cm/s.
- Straight line trajectory of 5 m with obstacle negotiation at the same speed as previous one.
- Ackerman steering of 180°, with radius 1.25 m. Rover linear speed at 1 cm/s.
- Turn-on-spot between 0° to 180° and *−*180° to 0° at 0.02 rad/s angular velocity.

### 6.2. Experimental Results

**Figure 9.**
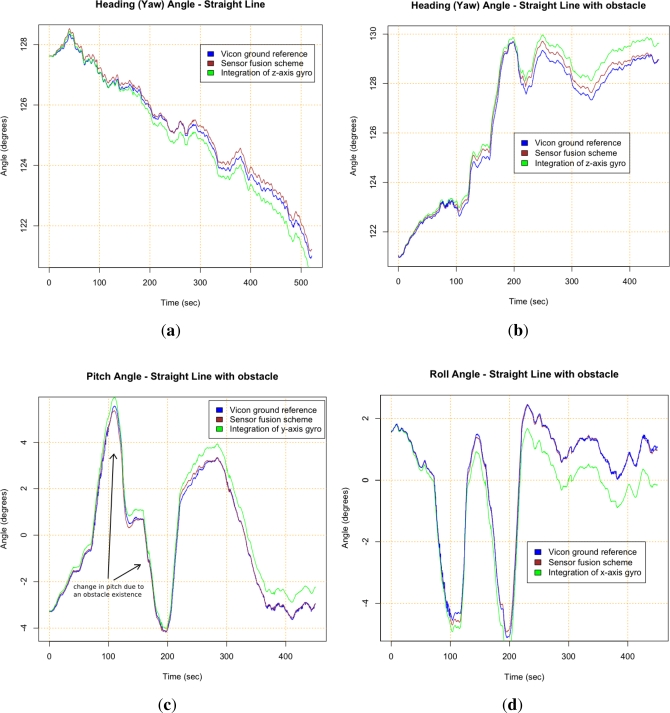
Results of the proposed approach for straight line tests. **(a)** Heading for straight path test; **(b)** Heading for straight path test with boulder negotiation; **(c)** Pitch for straight path test with boulder negotiation; **(d)** Roll for straight path test with boulder negotiation.

**Figure 10.**
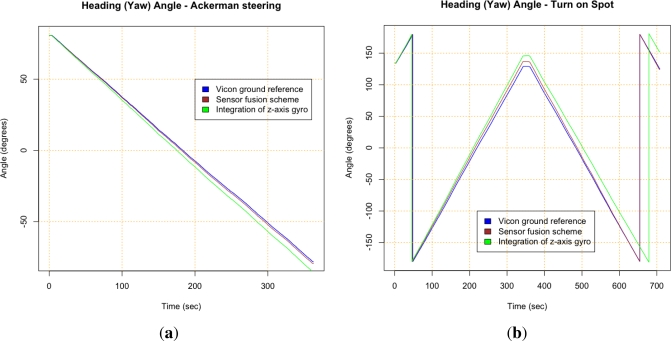
Results of the proposed approach for turning maneuvers as Ackerman and Turn-on-Spot. **(a)** Heading for Ackerman steering trajectory; **(b)** Heading for Turn-on-Spot test.

Results from the four test trajectories can be seen in Figures 9 and 10, where results of the Vicon system, the sensor fusion scheme and integration of gyros are compared. Integration of gyros are dominated by the drift. Table 6 also summarizes the maximum error between the Vicon reference measurement and the output of the scheme developed with the presented methodology (brown *versus* blue lines in figures). Figures 9(a) and 9(b) show that gyros bias instability is the main error for straight maneuvers as it was expected. Figures 9(c) and 9(d) depict good results for pitch and roll from the fusion of accelerometers and gyros using the QR estimator. Figure 9(a) and 10(a) show that results in the heading are better for the straight path than for the Ackerman steering. This is due to the fact that the scale factor error influences the measurement when measuring rate rotation. The scale factor error of the gyros was not possible to properly characterize since a rate-table was not available at the time of the error characterization tests. The results depicted in Figure 10(b) confirm this, indicating that the scale factor is the dominant gyro error for the sensor fusion scheme when the rover performs turning maneuvers (see Table 6).

## 7. Discussion and Future Work

An E2E methodology for increasing the performance of inertial sensors within a localization framework, with application to planetary rovers, has been presented. The approach has been validated with good test results of a new MEMS sensors on a representative space robotic platform. The applicability of the Allan Variance as a straightforward error characterization method and the importance of incorporating the characterization results in a sensor error model was demonstrated. In this line, an open source software package in R [21] has been developed in order to allow new tests in the future. The experimental tests onboard the LRM clearly showed that inertial sensor dominant errors are motion or trajectory dependent. In the case of the IMT30 it has been proved through experimental testing that the scale factor is one of the dominant errors in turning manoeuvres of the rover. This result highlights the importance of performing as complete error characterization as possible. Additionally it was observed that when the scale factor is not dominant, the reference measurement system error is in the order of magnitude of the sensor fusion scheme error (see Table 6). The Vicon system accuracy could be improved for future measurements by using more than the available eight cameras, but already the achieved accuracy is acceptable. On the positive side, the comparative magnitude of the Vicon measurement error to the sensor scheme error also demonstrates good performance of the presented methodology and the developed algorithms.

In the context of planetary rovers, the proper question would be whether this kind of lighter MEMS IMUs would be considered for future mission programmes. It is essential to understand how it will affect to the whole INS subsystem and consequently to mission operation. Exploration rovers in Mars set a baseline of error in heading and attitude angle within a range of two degrees after one sol (Martian day—24.6 h). Around 2–4 h traveling are typically considered depending on power availability and scientific interests. The distance traveled by current rover concepts is about 100 m per sol. Taking the values of the straight line test and supposing the Vicon system as a perfect truth, the pose error will be 3 cm after 5 m traveling. The error after 100 m would be less that one percent, which is in the range of the best performance for visual algorithms. Therefore, the accuracy of such inertial sensors tested on this work represents a precision enough to travel several meters without external correction using *tactical-grade* inertial sensors. Small correction would also be necessary from other localization techniques like visual odometry or a sun sensor, but the fact that the error from MEMS inertial sensors shows encouraging results may allow to design for visual corrections on the rover pose at low frequency rate. This is an advantage because the computational power is very limited in space avionics.

Attitude propagation of Mars Exploration Rovers (MER) [22] is computed by the Navigation/Attitude Estimator (NATE) [23] considering only gyros integration since the quality of the optic gyroscopes are good enough (≈1°*/h* drift was observed [23]), keeping the software simple. However, such *navigation-grade* IMU with more accurate sensors based on optic fiber technology are heavier, challenging the mass allocation and power consumption. The work presented in this manuscript demonstrates that nowadays good results can also be achieved with *tactical-grade* lighter MEMS inertial sensors properly characterizing and modeling the sensor noises and incorporating them in the sensor fusion design.

Future research will address the incorporation of a sun sensor to the sensor fusion scheme ending in a complete filter structure for the three angles with heading correction. The rover stops every few meters for path planning and obstacle mapping from navigation and hazards cameras, re-localization algorithm will be possible to implement in future developments when traveling longer distances. In addition, computational cost improvements of the presented approach will investigate the potential advantages of the Squared-Root Unscented Kalman Filter [24].

## Figures and Tables

**Table 1.** Gyroscope Thermal Drift.

**Coefficient**	**Gyro X**	**Gyro Y**	**Gyro Z**
Thermal drift [*^°^/s/^°^C*]	0.02	0.056	0.050

**Table 2.** Angle random walk noise coefficients for IMT30 gyros. Mean value of five tests.

**Coef.**	**Gyro X**	**Gyro Y**	**Gyro Z**
N	$0.20764{}^{\circ}/\sqrt{h}$	$0.23858{}^{\circ}/\sqrt{h}$	$0.20489{}^{\circ}/\sqrt{h}$

**Table 3.** Dominant stochastic errors in inertial sensors.

**Noise type**	**Allan variance***σ*^2^(*τ*)	**Coef.**	**PSD**
Quantization noise	$\frac{3Q^{2}}{\tau^{2}}$	*Q*[^°^]	(2*πf*)^2^*Q*^2^*τ*
Angle random walk	$\frac{N^{2}}{\tau }$	$N[{}^{\circ}/\sqrt{h}]$	*N*^2^
Bias instability	$\approx \frac{2B^{2}\text{ln}2}{\pi }$	*B*[°/*h*]	$(\frac{B^{2}}{2\pi })\frac{1}{f}$
Rate random walk	$\frac{K^{2}\tau }{3}$	$K[{}^{\circ}/h/\sqrt{h]}$	${\left(\frac{K}{2\pi }\right)}^{2}\frac{1}{f^{2}}$
Rate ramp	$\frac{R^{2}\tau^{2}}{2}$	*R*[°/*h*^2^]	$\frac{R^{2}}{{\left(2\pi f\right)}^{3}}$

**Table 4.** Rate random walk noise coefficients for IMT30 gyros. Mean value of five tests.

**Coef.**	**Gyro X**	**Gyro Y**	**Gyro Z**
K	$32.8{}^{\circ}/h/\sqrt{h}$	$49.6{}^{\circ}/h/\sqrt{h}$	$64.2{}^{\circ}/h/\sqrt{h}$

**Table 5.** Bias instability coefficients for IMT30 gyros. Mean value of five tests.

**Coef.**	**Gyro X**	**Gyro Y**	**Gyro Z**
B	3.8*°/h*	5.9*°/h*	5.3*°/h*

**Table 6.** Maximum attitude errors for the proposed fusion scheme during tests on the PUTB.

**Straight line**			**Ackerman steering**			**Turn on Spot**		
**Pitch**	**Roll**	**Yaw**	**Pitch**	**Roll**	**Yaw**	**Pitch**	**Roll**	**Yaw**
0.2^°^	0.1^°^	0.35^°^	0.3^°^	0.3^°^	1.4^°^	0.2^°^	0.4^°^	6.5^°^
